# Connection after correction: Parent‐child relationships following early cardiac surgery

**DOI:** 10.1002/imhj.70097

**Published:** 2026-05-15

**Authors:** Tamera A. Clancy, Frank Muscara, Carolina de Weerth, Brigid Jordan

**Affiliations:** ^1^ Department of Pediatrics University of Melbourne Melbourne Australia; ^2^ Brain and Mind Group Murdoch Children's Research Institute Melbourne Australia; ^3^ The Royal Children's Hospital Melbourne Australia; ^4^ Donders Institute for Brain, Cognition and Behaviour Radboud University Medical Center Nijmegen the Netherlands

**Keywords:** cardiac surgery, congenital heart disease, early life stress, emotional and behavioral regulation, emotional availability, infancy early childhood, longitudinal research

## Abstract

Early cardiac surgery for congenital heart disease (CHD) may have enduring effects on the parent‐child emotional connection and a child's capacity for regulation. This study explored emotional availability in parent‐child interaction following early cardiac surgery in relation to emotional and behavioral regulation in children and identified early predictors. Participants were 23 preschool‐aged children with CHD and their parents from a longitudinal cohort assessed at infancy (post‐surgery) and preschool age in Australia. Dyadic interaction was assessed using the Emotional Availability Scales (EA), and parents reported on child's emotional and behavioral regulation. Predictors included infant social‐emotional functioning, parental attachment feelings and traumatic stress, hospitalization length, timing of diagnosis and duration of contact at birth. Over 50% of dyads demonstrated emotional availability, however 30% were classified as complicated and 12% detached. Greater parental sensitivity was associated with less externalizing behavior. Longer hospitalization was associated with lower child involvement and greater parental intrusiveness. Contact at birth was associated with greater emotional availability and more optimal parental structuring at preschool. Findings highlight the enduring impact of early medical adversity on emotional availability in CHD and illuminate effects on both child and parent. Future research should explore targeted interventions that buffer relational disruptions in medically vulnerable dyads.

## 1 INTRODUCTION

Children who begin their lives with CHD requiring cardiac surgery often have challenges to their early regulation and then to later emotional and behavioral development. Given strong evidence that responsive caregiving, co‐regulation, and parental buffering can support healthy development in the face of early adversity (for a review, see Hostinar & Gunnar, 2015), it is essential to examine these relational processes within the context of CHD. The early experience of surgery and hospitalization for correction of CHD is a profoundly stressful and potentially traumatic experience for both the infant and their parent(s). Infants endure multiple stressors, including but not limited to, invasive medical and surgical treatments, frequent handling by unfamiliar hands manipulating their fragile body and sensory stimulation from bright lights, unusual smells, and strange sounds (Kazak et al.,^2005^). Infants with CHD may experience their body as unreliable and unpredictable, with these experiences potentially overwhelming their capacity to regulate, leading to negative emotionality, hyperarousal, or avoidance symptoms of a trauma stress response (Jordan et al., 2013).

Simultaneously, parents must navigate uncertainty and their own anxieties, engage in medical decision‐making, and provide supportive care for their sick child in a hospital environment that is tense and unfamiliar. These experiences can have pervasive effects, impacting not only the infant's physical body but also their social‐emotional wellbeing, compromising opportunities for parent‐infant bonding, and adversely impacting parent mental health and family functioning (Jordan et al., 2014). Considering 1.35 million babies worldwide are born with some form of CHD every year (van der Linde et al., 2011) and given the increased survival rates for children with CHD (Corno & Utens, 2018), it is imperative to better understand the child's individual needs but also the critical role of the emerging parent‐infant relationship (Browne et al., 2016) in shaping long‐term emotional and developmental outcomes.

There has been longstanding recognition of the impact of medical traumatic stress on children and parents impacted by pediatric illness (Kazak et al., 2005). Medically fragile infants exposed to significant stress and hospitalization early in life are at heightened risk for developmental delays (Oudgenoeg‐Paz et al., 2017), disrupted social‐emotional functioning and behavioral challenges (Vanderbilt & Gleason, 2011). Similarly, the unique burden and parenting demands arising from a child's CHD have consistently been shown to negatively influence the overall mental health and wellbeing of parents (Golfenshtein et al., 2017; Woolf‐king et al., 2017). Elevated parent distress within and beyond the perinatal period is associated with increased risk of emotional and behavioral difficulties in children with CHD (Visconti et al., 2002). Furthermore, untreated parental mental health symptoms influence parent‐child interaction, demonstrated in lower maternal responsivity and lower child positive interactivity (Sood et al., 2021). The way these children and their parent(s) adapt to stressful events likely plays an important role in the foundation of their resilience and the emotional and behavioral adjustment of children with CHD (Abda et al., 2018).

**Key findings**

- Most parent‐child dyads demonstrated strong emotional availability at preschool age, despite the significant medical and emotional stressors associated with early cardiac surgery.
- Higher emotional availability was characterized by greater parental sensitivity, responsive child engagement, and the absence of hostile or withdrawn caregiving behaviors.
- Early infant social‐emotional vulnerabilities and parent mental health symptoms were associated with lower emotional availability several years later.
- Findings underscore the importance of early identification of relational risk and the provision of relationship‐focused supports for families navigating medical adversity in infancy.

**Statement of Relevance**

As survival rates for infants with congenital heart disease (CHD) increase, understanding their developmental and relational trajectories has become increasingly important. This study broadens the CHD outcomes conversation beyond neurocognitive risk by centering the parent–child relationship. Findings show that many parent–child dyads demonstrate strong emotional availability despite significant early medical adversity, while early social–emotional vulnerabilities and parent mental health symptoms highlight opportunities for timely, relationship‐focused support within pediatric cardiac care.

Children with CHD face multiple early stressors, including pain, procedural interventions, and separation from parents, conditions that may increase their vulnerability to toxic stress and compromise the development of co‐regulatory processes (Jordan et al., 2013; Schore, 2001). Limited opportunities for parents and children to develop co‐regulatory strategies during hospitalization can influence a child's emotional and behavioral regulatory capacities, which are a necessary foundation for ongoing future development and wellbeing (Browne et al., 2016). The toxic stress model (Shonkoff et al., 2012) offers a useful framework for conceptualizing the cumulative impact of early medical adversity, such as cardiac surgery, on children with CHD. When a child's stress response systems are activated within an environment of supportive relationships with a parent, these physiological effects are buffered and return to baseline levels (Senehi et al., 2021; Shonkoff et al., 2012). The result is the development of healthy stress response systems. However, if the stress response is extreme, long‐lasting, and buffering relationships are unavailable to the child, the result can be toxic stress, leading to disruptions in self‐regulatory capacities (Hostinar et al., 2015; Shonkoff et al., 2012). Early childhood represents a period of plasticity, and this period may provide critical opportunities for prevention and early intervention (National Scientific Council on the Developing Child, 2007).

Because of illness and treatment factors associated with CHD, including hospitalizations, children with CHD may have limited access to or opportunity for contact and communication with their parent(s) during these stressful periods early in life. An infant uses behavioral cues (i.e., facial expressions, crying, tone of voice, and gesture) to communicate their emotional state to their caregiver, who then acts to regulate infant emotions through timely and accurate response to the infant's needs (Weinberg & Tronick, 1994). Early parent‐infant interactions serve a crucial function in scaffolding and supporting infants’ self‐regulatory experiences (Feldman, 2007), as infants rely heavily on their parents for external regulation of arousal and emotional distress (Spangler et al., 1994). For children with CHD, their early relational experiences and interrupted proximity with parents might impede or delay the development of emotion regulation and intersubjectivity that enables one to “read” other people or oneself (Bellinger, 2008; Trevarthen & Aitken, 2001) and compromise the infant's ability to effectively tolerate stressful experiences (Habersaat et al., 2014). Beyond the potential impact on emotional and behavioral regulation, the possibility to communicate through touch and physical availability, which has been shown to be crucial for the infant to obtain comfort, closeness, and protection (Field, 2010), is not readily available to the infant during hospitalization. Such experiences may limit both the infants in organizing functional responses and the parents in reading and appropriately interpreting the infants’ signals, which may impair their sensitivity and responsivity (Cassibba et al., 2012).

Emotional Availability (EA; Biringen, 2008) is a relationship construct that describes overall parent‐child interactional quality and focuses on two partner's accessibility to each other and their ability to read and respond to each other's communications (Biringen & Robinson, 1991). Attachment theory provides the foundation for the concept of emotional availability, sharing concepts of sensitivity and describing the emotional signaling that occurs between parent and child during interactions (Biringen et al., 2014). Emotional availability has been referred to as the “connective tissue” in parent‐child relationships, highlighting its role in affective exchanges throughout childhood (Easterbrooks & Biringen, 2000). In contrast to approaches focusing on discrete behavioral acts, the concept of dyadic emotional availability, as operationalized in EA, is global and emphasizes the reciprocal and transactional nature of the relationship (Biringen, 2008).

Through emotional responsiveness, the parent and child are continuously shaping and being shaped by each other's responses (Biringen et al., 2014), and the child is developing an understanding of their emotional states. The parent's consistent ability to accurately perceive, interpret, and accept their child's psychological state (Ainsworth et al., 1979) facilitates the child's emotional and behavioral regulation during distressing events (i.e., during vaccinations; Badovinac et al., 2018) and supports responsive and involving interactions. This is important in the context of CHD, given the suggestion that children born at high medical risk may benefit from parenting that is sensitive and non‐intrusive in timing and nature (Forcada‐Guex et al., 2006; Landry et al., 2006). Less sensitive, more disrupted interaction patterns might heighten the child's stress‐induced arousal and contribute to emotional and behavioral dysregulation.

Early signs of psychological distress for children with CHD have been demonstrated, with 3‐month‐old infants displaying pronounced negative emotionality (Torowicz et al., 2010) and parents describing their infant as difficult to soothe, irritable, and negative in mood (Jordan et al., 2013). Indeed, children who experience early medical adversity may display withdrawn, passive, or dysregulated interaction patterns, reducing opportunities for reciprocal parent‐infant engagement (Feldman, 2007; Montirosso et al., 2010). This is consistent with a temperament study, in which parents described their 4‐ to 8‐month‐old infants with CHD as intense, withdrawn and as having low thresholds for stimulation (Marino & Lipshitz, 1991). The presence of limits or distortions in the children's communicative behaviors due to illness may interfere with development of a secure attachment relationship (van IJzendoorn et al., 1992). Negative behavioral characteristics of the infant, such as being irritable and unadaptable, might make parenting efforts ineffective and contribute to parental distress and a feeling of not coping (Doherty et al., 2009; Solberg et al., 2011). Consistent with this, Torowicz et al. (2010) found infant emotional negativity and difficulty soothing explained more than 50% of the variance in maternal stress scores, even when controlling for stress related to infant medication, growth status, and hospital visits related to CHD.

The experience of having a child hospitalized due to a life threatening illness can lead to significant adverse psychological reactions in parents (Muscara et al., 2015). Persistent psychological distress experienced by parents from diagnosis, during birth, cardiac surgery, and beyond is well‐recognized by health professionals (Mangin‐Heimos et al., 2023; Uzark & Jones, 2003). Indeed, early research in the 1970s, revealed maternal anxiety was primarily a function of the presence of CHD, rather than condition severity (Kitchen, 1978). A review of the literature reveals parents of children with CHD experience more fear, anxiety, depression, hopelessness, and distress than parents of healthy children or those with other diseases (Lawoko & Soares, 2006). The shared experience of prolonged hospitalization increases parental stress, anxiety, inadequacy, disappointment and grief, and is almost certainly distressing for the child, impacting on their developing bond with their parent (Landolt et al., 2014; Lisanti, 2018). Qualitative studies across Australia and Canada reveal that parents identify separations during hospitalizations as a significant contributor to difficulties experienced bonding with their infant with CHD (Jordan et al., 2013; Rempel et al., 2013). A systematic review of prenatal and childhood studies found clinically significant levels of depression, anxiety, or posttraumatic stress disorder in 25%–50% of parents, with over 80% endorsing at least one trauma‐related symptoms (Woolf‐King et al., 2017). Similarly, Franich‐Ray et al. (2013) studied parents of infants who had cardiac surgery before 3‐months of age and found 34% of mothers and 18% of fathers met criteria for Acute Stress Disorder.

Parent mental health, coping skills, and family functioning are significant among parent factors which feed into the parent‐infant relationship, facilitating attunement in the short term and influential for long‐term health and well‐being (Murray & Cooper, 1997). If this relationship is compromised by stress and illness, opportunities for children to develop and practice affective self‐regulatory skills may be lost or diminished (Treyvaud et al., 2009). As Jordan et al. (2014) contend, a parent's psychological reactions and pre‐existing psychosocial vulnerabilities may impair their capacity to provide adequate buffering support for the stress experienced by their child during early cardiac surgery. In line with this, parental psychological distress may reduce a parent's emotional availability to assist their child in co‐regulating pain‐related distress (Lieberman, 2004). Increased stress in parents of infants with CHD has been associated to the child's psychosocial adjustment and behavior problems (DeMaso et al., 1991; Hearps et al., 2014). Parents with elevated psychological distress may be less attentive to their child's distress signals, less available to provide emotional support or even exhibit negative behaviors when their child experiences distress (Borelli et al., 2015; Moller et al., 2015). Given sensitive, responsive parenting and high‐quality relationships support the development of emotion regulation in children (Smith et al., 2006), it is conceivable that preschoolers with CHD who experience more emotionally attuned caregiving may exhibit a greater capacity to regulate emotion and behavior. Conversely, a distressed parent may not be able to adequately address their child's needs, which in turn may engender or reinforce behavioral problems in the child (Appleyard & Osofsky, 2003). Accumulating evidence demonstrates that psychosocial impairment is detectable early in infancy and that young children with CHD face elevated risks for emotional, social, and behavioral developmental difficulties (Clancy et al., 2020).

Despite recognition of the importance of early relational experiences (National Research Council et al., 2000), the quality of the parent‐child relationship in the context of CHD remains underexplored. Research has predominantly focused on medically modifiable factors, neurodevelopmental outcomes, and parent mental health. Few studies have explored the nature or trajectory of parent‐child interactions, nor have they identified early‐life factors that influence relationship outcomes following cardiac surgery. Of the research conducted, results have been mixed, finding both less sensitivity (Gardner et al., 1996) and more sensitivity (Harrison, 2013) in mothers of infants with CHD. Interestingly, mothers of infants with CHD scored significantly lower on a measure of fostering socio‐emotional growth, demonstrating they were less apt to smile, make eye contact, touch, hum or sing during a feeding interaction (Lobo, 1992). Goldberg et al. (1991) found infants 12–18 months of age with CHD were significantly less likely to have secure relationships with their mothers, in comparison to healthy peers, with markedly more avoidant attachment relationships.

Parental psychological wellbeing, particularly in neonatal and intensive care settings, is known to impact sensitivity and relational quality yet remain overlooked in CHD research (Woolf‐King et al., 2017). Although parental stress and depression is associated with poorer relational outcomes in CHD (Darke & Goldberg, 1994; Jordan et al., 2014), the role of posttraumatic stress symptoms remains unclear, and the influence of sociodemographic factors is similarly under‐investigated (Booth et al., 2018). Of the scant studies that have explored the parent‐child relationship in CHD, there have been varied methodologies with inconsistent application of constructs and interactional contexts assessed, limiting interpretability. This has contributed to mixed results regarding relational outcomes and has hindered understanding of the dynamics underpinning parent‐child interactions. Direct observations are considered invaluable for assessment of parent‐child interaction (Aspland & Gardner, 2003) and can be used to identify relationship risk as well as inform clinical intervention. Despite this, research efforts have relied on parental reports of relational dynamics, which may be shaped by reporting bias, reflective capacity, attachment representations, and parental distress related to their child's illness (Latva et al., 2008; Mikulincer & Florian, 1999).

A recent systematic review identified only six observational studies of parent‐child interaction in CHD (Tesson et al., 2021) and although there was no evidence of widespread disruptions in early relationships, results highlighted a proportion of dyads vulnerable to relational difficulties. In one of the first standardized observational measurements of infant social withdrawal following cardiac surgery, Re et al. (2018) revealed very high levels of maternal and infant distress, and a clear association between maternal distress and infant social withdrawal. Given the context of early cardiac surgery, relationship stress and disruption are unsurprising, and both infant responsiveness and parental emotional availability may be compromised. Further investigation is needed to explore this dynamic and investigate developmental consequences for the child.

### 1.1 The current study

This study addresses a key gap by examining the emotional availability of parent‐child dyads in the context of CHD and its associations with emotional and behavioral regulation at preschool age. A unique strength of this study lies in the use of direct observational measurement to assess parent‐child interactions, offering an objective, clinical researcher‐rated assessment of relational quality. Integrating observational and parent‐report data also enhances the validity of findings. By employing a multidimensional framework that captures both parent and child behaviors in interaction, the study advances understanding of the child's contribution to the relationship, in addition to the parents.

The longitudinal design, drawing on prospective data from infancy and informed by the Toxic Stress Model (National Scientific Council on the Developing Child, 2007), enables investigation into how early parental buffering and co‐regulation, particularly during critical periods of hospitalization and recovery, shape interactional quality and child regulation outcomes at preschool age. This developmental perspective offers critical insights into early risk and protective factors that may inform clinical practice to support child wellbeing and relational outcomes in the context of CHD. Limited research has examined parent‐child interaction quality in relation to the emotional and behavioral wellbeing of preschool‐aged children with CHD, and potentially modifiable predictors influencing co‐regulation and buffering support have not been elucidated.

We aimed to describe the emotional availability of parent‐child dyads following cardiac surgery for CHD in infancy and to examine associations with emotional and behavioral regulation at preschool age. A secondary aim was to identify early predictors of emotional availability in the preschool period, with a specific focus on early signs of infant distress and opportunities for co‐regulation and buffering support soon after initial cardiac surgery.

## 2 METHOD

### 2.1 Design

This is a prospective, cohort study with a longitudinal design, utilizing quantitative research methods including standardized, clinician‐rated observational measures, parent‐report measures, and medical data.

### 2.2 Participants

Participants were recruited from “*The Heart Supports Study*,” an investigation of the impact of cardiac surgery on children and parental adjustment. Infants were eligible if they had open or closed cardiac surgery for CHD within the first 6 months of life at the Royal Children's Hospital (RCH) Melbourne, Australia, from December 2014 to December 2015. Fifty‐three infants and their parents participated at Time 1, 6 weeks post‐discharge from surgery. At Time 2 (January–August 2019), 62% (33/53) preschooler‐parent dyads of the Heart Supports cohort participated, of whom, 23 participated in in‐person data collection. Children were between 3 and 4 years of age. Another 10 families provided questionnaire data at Time 2, although were unable to engage in the in‐person aspects due to geographical distance. Twenty families did not participate at Time 2 (unable to establish contact [*n *= 10], child died [*n *= 1]) and nine lost to follow‐up.

### 2.3 Procedure

At Time 1, parents completed the *Heart Support Study: Infant and Family Wellbeing’ Questionnaire*, 6 weeks after their infant (*M*_age_ = 4.8 months, SD = 1.8) was discharged from hospital following cardiac surgery.

At Time 2, participants and their parent(s) attended one laboratory visit for data collection purposes (duration 1–1.5‐h) at the RCH, where their early cardiac surgery occurred. The testing room was equipped with unobtrusive video recording equipment to allow for future coding of interaction. Parents also completed the Time 2 study questionnaire.

### 2.4 Measures

#### 2.4.1 Time 1

*Early Signals of Infant Distress*:
*The Ages and Stages Questionnaire: Social Emotional for Children* (ASQ:SE; Squires et al., 2002) is a 22‐item parent‐completed questionnaire that screens social and emotional problems in self‐regulation, compliance, communication, adaptive functioning, autonomy, affect and social interaction domains in children aged 3–60 months. Parents rate frequency of occurrence for each item with “most of the time,” “sometimes,” and “rarely or never,” and indicate whether the behavior is of concern. Total score was analyzed as a continuous variable and compared to the normative mean and standard deviation. Children who scored higher than the established cut‐off score of 45 for up to 6‐months of age (10% in the normed population) were classified as “at risk,” indicating further evaluation and/or intervention is required (Squires et al., 2002). An alpha coefficient of  .69 has been reported for the ASQ:SE, infant version (Squires et al., 2002).
*Illness/surgery factors and health status*: Infant medical data was obtained from the RCH medical database and parent‐report. Length of hospital stay (days) at the time of cardiac surgery was used as a proxy for the degree of stress experienced during hospitalization.

*Early opportunities for co‐regulation and buffering support*

1. *Parental traumatic stress* was measured using the Posttraumatic Stress Disorder Checklist for DSM‐5 (PCL‐5; Weathers et al., 2013), which is a widely used, 20‐item self‐report questionnaire to assess DSM‐5 symptoms of PTSD. Parents indicated how much they had been bothered by symptoms in the past month, on a 5‐point Likert scale ranging from 0 = *not at all* to 4 = *extremely*. Four PTSD symptom clusters are assessed: intrusion, avoidance, negative alterations in cognition and mood, and hyperarousal. Total scores range from 0 to 80, with higher scores indicating greater severity of PTSD symptoms. The PCL‐5 has high internal consistency (*α *= .94), test‐retest reliability (*r* = .82), and concurrent validity (*r* = .74–.85) (Blevins et al., 2015).
2. *Early parental attachment feelings* were measured using the Postnatal Attachment Scale (PAS, Condon & Corkindale, 1998), which is a 19‐item self‐report questionnaire of a parent's perception of their attachment to their infant in the post‐natal period. Items are rated on a 5 point scale, yielding total scores from 19 (low attachment) to 95 (high attachment). The PAS demonstrates good reliability (*α = *.79; Condon et al., 2008).
3. *Timing of Diagnosis* was included as a binary variable indicating whether the child's CHD was diagnosed prenatally or postnatally. This variable was selected to account for differences in parental preparation, stress, and early caregiving experiences.
4. *Contact at Birth*: To measure early opportunities for buffering support, parents reported how much time they spent with their baby immediately after birth. Responses were dichotomized to indicate whether contact lasted less than 10 or 10 min or more. This threshold was informed by previous research suggesting even brief early contact can meaningfully influence parent‐infant bonding and child emotional and behavioral regulation (Latva et al., 2008).

#### 2.4.2 Time 2

*Emotional and Behavioral regulation* at Time 2 was measured using the Child Behavior Checklist: 1.5–5 years (CBCL; Achenbach & Rescorla, 2000), a 99‐item norm‐referenced parent‐report questionnaire of child behavior in these domains: emotionally reactive, anxious/depressed, somatic complaints, withdrawn, sleep problems, attention problems, and aggressive behaviors. Frequency of behaviors is rated over the previous 2 months from 0 = “not true” to 2 = “very true or often true.” Externalizing scale (e.g., aggression, defiance, and conduct problem behaviors), internalizing scale (e.g., anxiety, depression, somatic complaints, and withdrawal behaviors) and total raw scores are calculated and transformed into standardized *T* scores (*M *= 50, SD = 10). The CBCL defines *T*‐scores ≥ 60 as representing a borderline clinical problem and *T*‐score ≥ 64 as clinically significant. Alpha coefficients for internal consistency have been reported as  .89 internalizing subscale,  .93 externalizing subscale, and  .95 total score (Achenbach & Rescorla, 2000). As a normative Australian sample has not been generated for this scale, published normative data of a United States of America sample was used for comparison (Achenbach & Rescorla, 2000).
*Quality of parent‐child interaction* was assessed using the *Emotional Availability (EA) Scales*, 4th Edition—Infancy/Early Childhood (Biringen, 2008). The EA is a global measure of dyadic emotional availability and indicator of the quality of the parent‐child relationship. For measurement using the EA, children and their parents were instructed to engage in free play for 20‐min with a standardized set of toys provided. The experimenter was not present for the parent‐child interaction. The videoed interaction was coded by researchers trained and certified as reliable by the developer of the EA coding system (Biringen, 2008). Inter‐rater reliability estimates were calculated as intraclass coefficients for the direct scores on each EA scale dimensions (sensitivity  .95, structuring  .92, non‐intrusiveness 1.0, non‐hostility  .95, responsiveness 1.0, and involvement  .95). The EA demonstrates strong construct validity and both short‐ and long‐term stability (Biringen et al., 2014).
The EA includes four adult (Sensitivity, Structuring, Non‐intrusiveness, Non‐hostility) and two child (Responsiveness, Involvement) dimensions. Each dimension was first directly scored using a 7‐point scale, from 1 (low) to 7 (high, optimal score) (Biringen, 2008). Next, an EA Zone (EA‐Z) score was calculated on a dimensional 1–100 global rating scale as a summary of the EA scales coded above, as well as to categorize EA into four distinct zones including: Emotionally Available, Complicated, Detached, and Disturbed/Traumatized (Senehi et al., 2021). For the parent‐child relationship to be scored in the Emotionally Available category (81–100), both parent and child are available and responsive to each other. In contrast, parent‐child dyads in the Complicated range (61–80) indicate incongruity in emotional availability and the Detached category indicates an emotional disconnection, but basic needs are met (41–60). The disturbed/traumatized category (0–40) indicates an extreme lack of parental sensitivity and child responsiveness (Biringen, 2008; Espinet et al., 2013). After scoring, EA‐Z scores were dichotomized (Senehi et al., 2021) to create two groups of high quality interaction EA‐Z = 1 (scores ≥ 81) and low quality interaction EA‐Z = 0 (scores ≤ 80). These dichotomized scores were used in mixed‐effects regression analyses.

## 3 STATISTICAL ANALYSIS

### 3.1 Data analytic plan

Data were analyzed using STATA Statistical Software Version 18 (StataCorp LLC., 2023). Demographic characteristics of the sample were summarized using means and standard deviations for continuous variables and frequencies (%) for categorical variables. Descriptive statistics are reported for all variables. Normality of continuous variables was assessed using Shapiro–Wilk tests and visual inspection of histograms and assumptions for parametric analyses were deemed appropriately met for all variables, except for EA‐Z.

We first conducted Pearson correlation analyses to examine associations between child emotional and behavioral outcomes (CBCL Total Problems, Internalizing, and Externalizing *T* scores) and emotional availability in parent‐child interaction (EAS subscales and EA‐Z). To examine predictors of emotional availability (EA‐Z) in parent‐child interactions at preschool age, we conducted bootstrapped ordinary least squares (OLS) regression analysis with 1000 replications to obtain robust standard errors, confidence intervals and *p*‐values. Bootstrapping was applied to address potential non‐normality in the dependent variable (EA‐Z), as indicated by a Shapiro–Wilk test (*p* = .052) and to improve estimate stability given the small sample size (*n* = 23). Standard OLS regression was used for other models where normality was not a concern. The predictor variables included: early signs of infant distress (ASQ:SE), Postnatal attachment feelings (PAS); Time held at birth (<10 min or ≥10 min); length of hospital stay (in days); parental traumatic stress (PCL‐5); timing of diagnosis (before vs. at or after birth).

To examine the association between these predictors and emotional availability dimensions within parent‐child interactions at preschool age, we conducted multiple linear regression analyses. Each regression model included a single subscale of the EAS at preschool age as the dependent variable. The distribution of length of hospital stay was skewed, so this variable was log‐transformed (ln) to approximate a normal distribution and allow for valid parametric analysis. All analyses were conducted on the full sample, including statistical outliers, to maintain data transparency and avoid selection bias. This approach preserved the integrity of the dataset and reflected the real‐world variability inherent in clinical samples, thereby informing valid and generalizable insights. To ensure robustness, sensitivity analysis were conducted. These included (1) robust regression to reduce the influence of extreme values and (2) analysis excluding outliers. A *p* value of less than  .05 was considered statistically significant.

## 4 RESULTS

### 4.1 Participant characteristics

The “*Heart Supports Study: Infant and Family wellbeing*” included 53 infants and parents who provided parent‐report questionnaire data at Time 1. This follow‐up study, “*Heart Supports: Life as a preschooler*” (Time 2), recruited 23 of those children (*M*_age_ = 47.48 months, SD = 3.19). Participants at Time 2 did not differ from dyads who participated at Time 1 on measures of parent age or child sex, CHD severity, or length of hospital stay (Table 1).

**TABLE 1:** Demographic characteristics of the Heart Supports Cohort: Comparison of participants at Time 1 and Time 2.

	Time 1 cohort *n* = 53	Time 2 cohort *n* = 23	Chi square (df), *p* value
Child age^a^ *M* (SD)	4.8 (1.85)	47.48 (3.19)	–
Child sex (female) *n* (%)	26 (49.1)	13 (56.5)	*χ* ^2^(1) = .91, *p* = .34
Prenatal CHD diagnosis *n* (%)	39 (73.6)	19 (82.6)	*χ* ^2^(1) = 1.70, *p* = .19
CHD severity^b^			*X* ^2^(1) = .15, *p* = .70
Acyanotic *n* (%)	20 (37.7)	8 (34.8)	
Cyanotic *n* (%)	33 (62.3)	15 (65.2)	
Neonate at time of surgery^c^ yes *n* (%)	37 (69.8)	15 (65.2)	*χ* ^2^(1) = .41, *p* = .52
Length of hospital stay (days) *Mdn* (range)	17 (5–110)	18 (5–110)	*z *= –1.03, *p *= .31^d^
Contact at birth (Less than 10 min), *n* (%)	41 (77.4)	18 (78.3)	*χ* ^2^(1) = .02, *p* = .89
Parent age (years) *Mdn* (range)	32.9 (22–44)	32.1 (26–44)	*z *= 1.24, *p *= .22
Biological parent of child *n* (%)	52 (98.1)	22 (95.7)	*χ* ^2^(1) = 1.33, *p* = .25
Parent country of birth: Australia (%)	41 (77.4)	19 (82.6)	*χ* ^2^(10) = 9.46, *p* = .49
Parent education: Tertiary level *n* (%)	27 (50.9)	14 (60.9)	*X* ^2^(1) = .01, *p *= .91

^a^ Age measured in months.

^b^ Cyanotic and acyanotic refer to physiological classification of CHD based on oxygenation. Cyanotic CHDs involve reduced oxygen in the bloodstream due to structural defects, often resulting in more severe symptoms and requiring more complex clinical management (Hoffman & Kaplan, 2002).

^c^ Neonate (defined as <28 days old) at time of surgery.

^d^ *z = *non‐parametric Wilcoxon rank‐sum test.

### 4.2 Descriptive analyses

#### 4.2.1 Early signs of infant distress

The first cardiac surgery for all infants in this cohort occurred prior to 6 months of age, with an average age of 30 days‐old (Table 2). The length of hospital stay varied, ranging from 5 to 110 days, with a median hospitalization duration of 18 days. At Time 1, 22% (5/23) children had ASQ:SE scores above the clinical cut off indicating social‐emotional difficulties of clinical interest.

**TABLE 2:** Descriptive statistics of Time 1 variables and the quality of parent‐child interaction and emotional and behavioral outcomes at Time 2 (*N *= 23).

Variable	*M*	SD	Range
Time 1			
Social‐emotional functioning^a^			
Total score	25.79	21.02	0–70
Parental attachment feelings^b^			
Total score	82.96	8.11	63–93
Parental traumatic stress^c^			
Total score	10.54	8.63	0–30
Time 2			
EA subscales^d^			
Adult sensitivity	5.41	1.11	3.5–7
Adult structuring	4.96	1.18	3–7
Adult non‐intrusiveness	5.59	.81	4–7
Adult non‐hostility	6.35	.82	4.5–7
Child responsiveness	5.35	1.02	3.5–7
Child involvement	5.35	1.08	3.5–7
EA‐Z^e^	*n*	(%)	
Emotionally available (81–100)	13	56.52	–
Complicated (61–80)	7	30.43	–
Detached (41–60)	3	13.04	–
Problematic (0–40)	0	0	–
CBCL^f^	*M*	SD	Range
Total problem *t*‐scores	49.87	9.77	34–66
Internalizing *t*‐scores	50.78	9.87	37–65
Externalizing *t*‐scores	48.52	9.83	32–67

^a^ ASQ:SE: Ages and Stages Questionnaire (range 0–80), measured at Time 1.

^b^ PAS: Postnatal Attachment Scale (range 0–95), measured at Time 1.

^c^ PCL‐5: Parental traumatic Stress (range 0–80), measured at Time 1.

^d^ EA subscales: Emotional Availability Scale scores (range 1–7) measured at Time 2.

^e^ EA‐Z: Emotional Availability Zone score (0–100), measured at Time 2.

^f^ CBCL: Child Behavior Checklist, Preschool version (range 0–100), measured at Time 2.

#### 4.2.2 Early opportunities for co‐regulation and buffering support

Most parents received their infant's diagnosis of CHD prenatally (82%), with 15 (65%) diagnosed at the 20‐week ultrasound scan. At birth, most parents (78.26%) had less than 10 min of direct contact with their newborn. Following their infants hospital discharge for cardiac surgery, three parents (9%) met DSM‐V diagnostic criteria for post‐traumatic stress disorder. At Time 1, parents reported strong attachment feelings toward their infant (*m *= 82.96, SD = 8.11), comparable to the Australian community mean for mothers of 4‐month‐old infants (*M *= 84.6, SD = 7; Condon & Corkindale, 1998). This difference was not statistically significant, *t* (22) = –0.97, *p *= .34. Ten parents (30%) had scores lower than 1 SD below the Australian community mean, which has been considered an indicator of “low attachment feelings” of clinical interest, as this score is lower than 85% of an Australian community sample (Jordan et al., 2014).

#### 4.2.3 Parent‐child interaction quality at Time 2

Over half of the dyads scored within the emotionally available range (56.52%) on the EA‐Z, with 30.43% of dyads in the complicated range and 13.04% in the detached range (Table 2). Average scores on non‐intrusiveness and non‐hostility subscales indicated most parents were within the optimal range. Parent sensitivity was moderately optimal and the lowest mean raw score was for the parent structuring subscale. Child dimensions of responsiveness and involvement of their parent were moderately optimal.

#### 4.2.4 Child emotional and behavioral regulation in association with emotional availability in dyadic interaction

In this cohort, parents generally reported that their preschool‐aged child displayed emotional and behavioral regulation within the average range, comparable to the normative population. However 26% of preschoolers (*n* = 6) had elevated scores which reached or exceeded the borderline/clinical cut off for internalizing behavioral difficulties and total problems. Externalizing behavior scores were elevated for 8.7% (*n *= 2) of preschoolers.

Child externalizing behavior was negatively associated with emotional availability within parent‐child interaction (*p *= .04) and higher parental sensitivity was significantly associated with lower externalizing behavior in children (*p *= .04; Table 3). Although parent structuring showed a negative association with externalizing behavior (*p *= .09), this did not reach statistical significance. Our analyses did not reveal any significant associations between EAS subscales and internalizing behavior difficulties or total problem scores.

**TABLE 3:** Associations between emotional availability and child emotional and behavioral regulation.

CBCL	EA parent subscales				EA child subscales		EA‐Z
	Sensitivity	Structuring	Non‐intrusiveness	Non‐hostility	Responsiveness	Involvement	
Externalizing behavior	**–.43** ^*^	–.36	–.14	–.40	–.36	–.25	**–.42** ^*^
Internalizing behavior	–.10	.26	.11	–.09	.05	–.03	–.05
Total problems	–.32	–.12	.02	–.28	–.20	–.19	–.26

*Note*: Pearson correlations shown. Significant *p* values are indicated by ^*^ *p* < .05 and shown in bold typeface.

### 4.3 Primary analyses

#### 4.3.1 Early signs of infant distress and opportunities for coregulation and buffering support as predictors of emotional availability within parent‐child relationships at preschool age

Early signs of infant distress accounted for 13% of the variance in emotional availability within the parent‐child relationship at Time 2 (Table 4). Higher infant social‐emotional difficulties were associated with lower emotional availability in parent‐child relationships at preschool age, this association did not reach statistical significance (*β *= –.19, *p *= .09).

**TABLE 4:** Bootstrapped regression analyses predicting emotional availability (EA‐Z) in parent‐child relationships at preschool age.

EA‐Z^a^	B (SE)	*z*	*p*	95% CI
ASQ:SE	–.19 (.11)	−1.76	.09	–.42, .04
PAS	–.35 (.25)	−1.38	.17	–.85, .15
Contact at birth	10.07 (3.31)	3.04	**.002** ^*^	3.58, 16.55
Length of hospital stay	−8.24 (2.90)	−2.84	**.005** ^*^	−13.93, –2.55
Parental traumatic stress	.14 (.25)	.55	.58	–.35, .63
Timing of diagnosis	8.45 (5.04)	1.68	.09	−1.43, 18.33

*Note*: ^a^ EA‐Z = Emotional Availability Zone; Bootstrapped standard errors, confidence intervals (CI) and *p* values obtained using 1000 replications; B = unstandardized regression coefficient; SE = bootstrapped standard error; *z* = *z* score, CI = 95% confidence interval. ASQ:SE: ages and stages questionnaire: social‐emotional development; PAS: Postnatal Attachment Scale; Contact at birth: >10 min; Length of hospital stay (in days); Parental Traumatic stress (PCL‐5); Timing of diagnosis (before & at/or after birth); Significant *p* values are indicated by

^*^ *p* < .05 and shown in bold typeface.

Our analysis revealed a significant positive association between contact at birth and emotional availability in parent‐child relationships at preschool age (*p* = .002). The observed pattern showed more contact at birth enhanced emotional availability in preschool years. Consistent with this, results also showed parents who had contact with their baby for more than 10 min demonstrated higher, more optimal, structuring scores (*B* = 1.21, *p* = .03), accounting for approximately 18.67% of the variance.

Longer periods of hospitalization at the time of cardiac surgery were associated with lower emotional availability at preschool age (*p *= .005). There was also a negative association (*p *= .04) between hospital stay duration and the child's involving behaviors toward their parent at preschool age, explaining 17% of the variance. Sensitivity analyses confirmed the robustness of these associations and excluding the outlier (hospital stay of 110 days) yielded similar results (*B* = –8.11, 95% CI [–15.32, –.91], *p *= .03).

Results indicated a negative association between length of hospitalization and parent's ability to remain non‐intrusive during interactions with their preschooler (*B *= −.06, 95% CI [−.11, −.01], *p* = .03) accounting for 18% of the variance, with longer hospital stays correlating with increased parental intrusiveness. A negative association between longer hospital stays in infancy and increased parental hostility explained 15.78% of the variance. While this relationship was not statistically significant (*B *= −.014, 95% CI [−.03, −.00], *p* = .07), these findings could have practical implications in clinical settings. Emotional availability was higher for dyads in which the CHD diagnosis was made postnatally, compared to those diagnosed prenatally, although this increase did not reach statistical significance (*p* = .09). No significant associations were found between parental traumatic stress or attachment feelings at Time 1 and emotional availability at Time 2.

## 5 DISCUSSION

This study provided novel insights into the emotional availability of parent‐child dyads in the context of CHD and explored associations with emotional and behavioral regulation in preschoolers who experienced cardiac surgery early in life. Through exploration of early signs of infant distress and opportunities for co‐regulation and buffering support utilizing prospective parent‐report data and observational methods, we identified predictors of the quality of the parent‐child relationship at preschool age, with important clinical implications.

### 5.1 Emotional availability in the CHD context

Emotional availability and relational strengths were evident in the interactions of preschoolers with CHD and their parents, following the experience of cardiac surgery early in life. Over half of the dyads displayed emotionally available relationships, demonstrated by parental sensitivity and responsivity to the child's emotions, needs and goals, and by the child's reciprocal engagement and responsiveness, suggesting predominantly positive and attuned relational functioning within the dyad. Most parents were observed to be within the optimal range for non‐intrusiveness and non‐hostility domains. Many parents had a non‐interfering presence in interaction with their child, characterized by a lack of over‐direction or stimulation and limited verbal or physical interferences. Of concern, nearly one‐third of dyads (30%) in our sample were rated within the Complicated range of emotional availability and a further 12% were rated as Detached. These classifications represent meaningful relational disruptions such as inconsistent, incongruent, or misattuned parental responses to the child's cues, low levels of reciprocity or emotional disconnection during interactions (Biringen et al., 2014). These subsets of dyads may be at elevated risk for ongoing difficulties in co‐regulation, emotional connection, and behavioral adjustment, highlighting the importance of early identification and relationally focused support.

Parent sensitivity, characterized by attunement, warm affect, and timely responsiveness, was moderately optimal, indicating most parents create a positive environment and form a healthy emotional connection with their child but with inconsistent or apparent sensitivity. In contrast, the lowest average score was for parent structuring, indicating parents were not providing guidance and scaffolding to optimally meet their child's needs. These findings are consistent with prior work showing compromised dyadic interactions with parents of children with CHD demonstrating low sensitivity to child cues (Gardner et al., 1996) intrusiveness, overcontrol, and high negative affect (Laing et al., 2010), lower attunement and reduced dyadic synchrony (Lobo, 1992). This may reflect the ongoing emotional and cognitive demands of parenting a medically fragile infant, as well as possible effects of early stress on both parent and child regulatory systems.

Child responsiveness to and involvement of their parent were moderately optimal, indicating some initiation to connect, engage and be responsive to their parent, though not as consistently or actively as a child in the optimal range. This aligns with studies demonstrating less social connectedness (Laing et al., 2010), less involvement, persistence and positive experience (Goldberg et al., 1990) and less responsiveness (Peçanha et al., 2015) in children with CHD aged 2.5–4 years. In Gardner et al.’s (1996) seminal observational study, infants who underwent cardiac surgery had difficulty sustaining engagement with their mother, while many mothers had significant difficulty adapting to these interactional disruptions. This led to low positive engagement and disordered interactions, which persisted at 6‐month follow‐up (Gardner et al., 1996). Collectively, these findings highlight the bidirectional, dynamic nature of early interactions, with relational difficulties stemming from both child and parent.

### 5.2 Emotional availability and emotional and behavioral outcomes at preschool age

Children in more emotionally available dyads, characterized by greater parental sensitivity, displayed less overt behaviors such as aggression and hyperactivity. Parental structuring also emerged as important, with scaffolding and organizing of interaction associated with greater behavioral regulation. These findings suggest that attuned, responsive parenting and developmentally appropriate guidance for children exposed to early stress from cardiac surgery may support improved behavioral regulation, echoing theories of co‐regulation and buffering support. Considering the reciprocal nature of relational adaptation, it is also possible that children with fewer regulatory challenges elicit more sensitive and attuned parenting (Feldman, 2012). Conversely, neurodevelopmental compromise secondary to CHD severity may reduce a child's responsiveness and social engagement, inadvertently eliciting more intrusive of over‐directive parental behaviors. This bidirectional perspective underscores the complex interplay between child neurodevelopmental vulnerability and dyadic emotional availability in shaping behavioral adjustment.

### 5.3 Early experience of cardiac surgery for CHD and emotional availability in the preschool period

Early postnatal contact is a fundamental component of postnatal care and plays a considerable role in facilitating bonding and supporting infant regulation and parent‐infant attachment (Nyqvist et al., 2010; WHO, 2003). In our study, the majority of dyads had less than 10 min together following birth, with this deviation from the normative birth experience potentially influencing parent‐child relationship quality overtime. Indeed, longer duration of early contact was associated with greater emotional availability and more optimal structuring at preschool age. While this may reflect the benefits of early bonding, it may be associated with differences in illness severity, as infants requiring urgent surgery have less opportunity for early contact. Therefore, this should be interpreted cautiously recognizing that medical necessity may drive early challenges.

Optimal structuring provides clear, developmentally appropriate boundaries and guidance while also promoting the child's sense of control and emotional safety (Biringen, 2008). This kind of supportive scaffolding is particularly important in healthcare contexts, where stress and uncertainty may otherwise overwhelm a child's capacity for emotional and behavioral regulation. Structuring is understood to foster both emotional security and behavioral organization in children and is considered foundational to high‐quality, parent‐child interaction (Biringen, 2008). These findings highlight the lasting impact of early contact on relationship quality and underscore the importance of facilitating opportunities for early bonding through parent‐infant contact and connection, particularly in the context of CHD, where standard postnatal experiences may be disrupted. This may serve to enhance the quality of the relationship, promote parental competencies and foster more attuned behavior toward the infant.

Prolonged hospitalization was associated with significant reduction in preschooler's ability and willingness to actively engage their parent in interaction. This aligns with existing evidence that early medical adversity, particularly extended hospitalization, disrupts social and emotional development, contributing to later difficulties in relational engagement and responsiveness. Consistently, early infant distress, measured by social‐emotional difficulties, was associated with lower relationship quality at preschool age. These findings align with developmental models suggesting early relational disruptions (i.e., infant distress or parental stress) impact co‐regulation and dyadic emotional availability (Feldman, 2007; Spangler et al., 1994; Sroufe, 2005).

In addition to the effects on the child, length of hospitalization was significantly associated with parental interaction patterns, particularly intrusiveness. Parents whose infant experienced prolonged hospitalization demonstrated greater difficulty in maintaining balanced and attuned interactions, potentially reflecting heightened anxiety or hypervigilance around caregiving, following an extended period of medical uncertainty, separations, altered routines, and restrictions on holding their infant. Although the association between hospitalization and parental hostility was not significant, results suggest prolonged stay may subtly alter dyadic interaction, possibly increasing covert parent frustration or withdrawal. The cumulative burden of stress, grief and heightened emotional demands may impact parent's capacity to provide sensitive, attuned interactions. Longer stays also likely reflect higher medical complexity and possible neurodevelopmental compromise, which may reduce infant's capacity for reciprocal interaction and, in turn, heighten parental anxiety or over‐vigilance. The relationship between medical severity, child responsiveness, and parental behavior is therefore likely multi‐directional.

Interestingly, Allen et al. (2004) demonstrated that length of hospitalization was associated with higher parental perception of child vulnerability among premature infants, suggesting length of stay was much more salient to parents than other indicators of neonatal illness severity. Extending on this, prolonged hospitalization has been theorized to reduce parents’ ability to be present and to identify, prioritize, and appropriately respond to their child's need for emotional co‐regulation (during a medical treatment) (Brown et al., 2018). As Wernovsky and Licht (2016) contend, although some aspects of length of stay may not be modifiable, contact with parents is a potential protective and modifiable factor. In the context of early, multiple and often prolonged separations during the time of surgery and hospitalization, it is imperative that opportunities for co‐regulation and buffering support during these experiences are considered and investigated as potentially impacting the developmental outcomes of children with CHD.

The experience of CHD early cardiac surgery, resultant stress and trauma are hypothesized to effect parent's ability to be emotionally available and sensitive to the child's needs, in turn influencing the child's emotion regulation and adjustment (Gardner et al., 1996; Samuelson & Cashman, 2008). However, we did not observe a significant association between parental trauma symptoms in infancy and later dyadic outcomes. One explanation may relate to the specific focus of our measure, which primarily captured symptoms of posttraumatic stress disorder. This may not be as sensitive to subtle forms of relational strain or emotional and behavioral dysregulation that might emerge from symptoms of depression and anxiety which have been more consistently linked to parenting behaviors/attachment in past research (e.g., Jordan et al., 2014). Additionally, it is possible that protective factors within our sample, such as effective coping strategies, access to social support and hospital or community based‐mental health services, may have mitigated the effects of early distress on later parent‐child relationships. Alternatively, the lack of observed association may reflect limitations in statistical power due to the small sample size. These findings highlight the need for future research to examine a broader range of parental emotional experiences using multimethod longitudinal designs to better understand how different forms of distress and resilience shape early relational outcomes in the context of CHD.

### 5.4 Clinical implications

Supporting emotional, behavioral, and relational needs is essential for promoting optimal outcomes for children who have experienced early medical adversity. Relational challenges may be more likely when parents receive a prenatal diagnosis, have limited contact with their baby at birth, or when infants with CHD show early signs of distress and experience prolonged hospitalization. Prenatal diagnosis may correspond with more severe or complex CHD, which may influence both parental stress and infant regulatory capacities. These risk factors present a critical opportunity to offer timely psychosocial clinical responses, and our findings highlight several targets for early intervention.

Prenatal diagnosis provides a valuable opportunity for the provision of support, psycho‐education, and to commence ongoing monitoring. These efforts may help empower parents, enhance coping and optimize developmental outcomes (Ryan et al., 2019). Our findings highlight the importance of longer immediate postnatal contact to support early parent‐infant bonding, even within the obstetric, surgical and intensive care context. Psychoeducational programs for parents and hospital staff targeting relational attunement may be beneficial in mitigating potential disruptions to emotional availability within the parent‐child relationship, simultaneously enhancing parental confidence and understanding of their child. Attachment‐informed and trauma‐sensitive interventions could be investigated to promote co‐regulation and sensitive responsivity. The “Close Collaboration with Parents” intervention by Ahlqvist‐Björkroth et al. (2025) offers compelling evidence of the benefits of increased parental presence and contact with their infant, improved infant growth and shortened length of stay through facilitation of family‐centered care in neonatal intensive care environments.

Approaches such as these align with infant mental health principles, highlighting the role of responsive relationships in buffering early stress and fostering healthy development in vulnerable infants (Browne et al., 2016; Lakatos et al., 2019). Interventions must extend beyond supporting the individual (infant or parent) to foster the developing parent‐infant relationship (Lakatos et al., 2019). Immediate and proactive clinical psychosocial intervention has the potential to support this relationship early in the CHD journey and to become an important part of the child's broader CHD treatment, mitigating the well‐documented vulnerability for long‐term behavioral, social and emotional difficulties (Shillingford et al., 2008). Emerging evidence also suggests that high‐quality caregiving may positively influence not only psychosocial outcomes but also physical health in children (Ehrlich & Cassidy, 2021; Stern et al., 2020), although this has not yet been directly investigated in CHD populations. Importantly, intervention efforts must account for medical severity and child neurodevelopmental status, acknowledging that relational processes occur within the context of biological vulnerability.

### 5.5 Strengths and limitations

While prior studies have examined the effects of CHD and cardiac surgery on behavioral and emotional outcomes, few have explored the impact on children at preschool age, and very few have examined associations with the quality of the parent‐child relationship. This study contributes to this gap by using standardized, observational, clinician‐rated measurement of emotional availability rather than reliance on parent report. Additionally, our findings provide a longitudinal perspective by linking very early social‐emotional difficulties with later relationship dynamics. However, the small sample size limits generalizability and statistical power to detect effects. Type and severity of CHD were not differentiated and may confound observed associations. Because the sample included a range of cardiac diagnoses and surgical complexities, the findings should be interpreted in light of this heterogeneity. More severe conditions, requiring immediate intervention or longer hospital stays, may themselves contribute to altered parent–child interactions and infant regulatory outcomes.

Although not statistically significant, associations between emotional availability and both early social‐emotional distress and timing of CHD diagnosis approached significance. This suggests these early experiences may play a role in shaping later parent‐child relational quality and highlight the importance of continued investigation with larger samples to clarify these associations.

Only a subset of the original cohort completed the observational task. Despite analyses showing no significant demographic or clinical differences between those who did and did not participate, it is still possible that those who engaged in this study were more motivated or higher functioning, potentially biasing the results. Contextual factors, such as stress, fatigue or family functioning which may influence parent‐child relationship quality, were not assessed as part of this study. Emerging evidence suggests that stress‐related experiences may be reflected not only in interactional patterns but also in biological responses such as cortisol reactivity (i.e., Hostinar & Gunnar, 2013). Research integrating behavioral and biological data may provide a more comprehensive understanding of co‐regulation, adaptation and resilience processes in families of children with CHD.

## 6 CONCLUSIONS

With the increased recognition of the potentially traumatic impact of CHD and early cardiac surgery on both children and their parents, there is a critical need for interventions that support the infant, the parent, and the evolving parent‐child relationship to foster resilience. This study contributes to this advancement by examining child emotional and behavioral outcomes in relation to the quality of the parent‐child relationship. A multidimensional approach was used, incorporating reliable and validated tools, specifically parent‐report questionnaires and clinician‐rated observational measurement. While overall emotional availability was evident in most dyads, specific relational challenges emerged, particularly inconsistent or misattuned parental responses, low levels of reciprocity, and emotional disconnection in interaction. Notably, more than 40% of dyads had complicated or detached relationships. Importantly, limited early contact and longer hospital stays were associated with less emotional availability at preschool age, though these factors likely reflect both relational and medical influences. Together, these findings highlight the lasting impact of early medical adversity on the parent‐child relationship and underscore the need for early infant and relationship‐focused support for families navigating cardiac care.

## CONFLICT OF INTEREST STATEMENT

The authors declare no conflicts of interest.

## Data Availability

The data that support the findings of this study are available on request from the corresponding author. The data are not publicly available due to privacy or ethical restrictions.
